# The Effect of Direct Anticoagulant Therapy on Haematological Parameters in Atrial Fibrillation: Clinical Significance of Subclinical Haemoglobin Decrease

**DOI:** 10.3390/medicina60111851

**Published:** 2024-11-11

**Authors:** Metin Çoksevim, İdris Buğra Çerik, Ömer Kertmen, Göksel Dağaşan, Murat Eroğlu, Ufuk Yıldırım

**Affiliations:** 1 Department of Cardiology, School of Medicine, Ondokuz Mayıs University, 55200 Samsun, Turkey; ufukyildirim2715@yahoo.com.tr; 2 Department of Cardiology, School of Medicine, Ordu University, 52200 Ordu, Turkey; cerikbugra@gmail.com; 3 Department of Cardiology, School of Medicine, Amasya University, 05100 Amasya, Turkey; omerkertmen@gmail.com; 4 Department of Cardiology, Alanya Training and Research Hospital, 07400 Antalya, Turkey; gokseldagasan@yahoo.com; 5 Department of Cardiovascular Surgery, Evliya Çelebi Training and Research Hospital, Kütahya Health Sciences University, 43020 Kütahya, Turkey; murateroglu5961@hotmail.com

**Keywords:** atrial fibrillation, direct oral anticoagulants, haemoglobin decrease, anaemia

## Abstract

*Background and Objectives*: Direct oral anticoagulants (DOACs) have become the cornerstone of stroke prevention in the management of atrial fibrillation (AF). While their efficacy in preventing catastrophic outcomes is well documented, the exploration of their effects on haematological parameters, particularly in clinically stable AF patients, remains markedly underrepresented in existing research. The aim of our investigation was to delineate the variations in key haematological parameters, with a special focus on haemoglobin (Hb), in a cohort of clinically stable patients afflicted with AF and receiving diverse oral anticoagulant treatments. *Materials and Methods*: In this retrospective study, 742 patients with AF were evaluated. Following exclusion criteria, 530 patients were included and categorised based on the change in their Hb levels (ΔHb < 2 [*n* = 473] vs. ΔHb ≥ 2 [*n* = 57]) after one year of initial prescription of DOACs. *Results*: Patients in the ΔHb ≥ 2 g/dL group demonstrated significantly higher baseline haemoglobin levels during the pre-DOAC period (13.5 [12.3–14.6] vs. 14.6 [13.1–15.7]; *p* = 0.002). Baseline haemoglobin was identified as a predictive factor for a decrease in Hb ≥ 2 g/dL, with higher initial values being associated with more pronounced reductions (OR, 95% CI: 1.424 [1.178–1.723]; *p* < 0.005). This pattern was observed consistently across various types and dosages of DOACs. *Conclusions*: This study underscores the importance of vigilant clinical monitoring for anaemia in patients undergoing DOAC therapy, even when their clinical course appears to be stable.

## 1. Introduction

Atrial fibrillation (AF) is a common arrhythmia that significantly increases the risks of stroke, systemic embolism, and death [1]. The incidence of stroke in patients with AF is five times higher, and the risk of mortality is nearly double that in individuals with normal sinus rhythm (NSR) [2]. The pathophysiology of AF is closely linked to atrial cardiomyopathy, characterised by structural and functional changes in the atrial myocardium that predispose individuals to arrhythmia and contribute to thromboembolic events. The development of cardio-embolic disease, a major complication of AF, underscores the essential need for effective preventive strategies to mitigate the associated stroke risk. The cornerstone of preventing stroke in the management of AF is oral anticoagulation, where direct oral anticoagulants (DOACs)—Factor Xa inhibitors (rivaroxaban, apixaban, and edoxaban) and direct thrombin inhibitors (dabigatran)—offer advantages such as fewer drug interactions and no need for routine monitoring compared to traditional vitamin K antagonists (VKAs) [3,4].

Despite extensive research on the bleeding risks associated with anticoagulation in AF, the nuanced bleeding patterns and the impact of subclinical haematological changes remain underexplored [4]. Notably, anaemia in patients with AF is strongly associated with adverse outcomes; however, the occurrence and management of subclinical haematological changes during anticoagulant therapy without overt bleeding signs are not well documented [5].

In this context, our study aimed to investigate the changes in basic haematological parameters in patients with AF initiated on different oral anticoagulant therapies and followed up stably, particularly to determine whether anaemia developed during follow-up, even in the absence of detectable bleeding.

## 2. Methods

In this retrospective analysis, we evaluated 794 patients diagnosed with AF at our centre who received DOACs for the first time. Based on the exclusion criteria, 530 patients were included in the study. The study spanned from April 2020 to September 2021, and the study included only those patients who completed a continuous 12-month follow-up from the date of their initial DOAC prescription, ensuring that each participant’s data reflected a full year of therapy. Medication adherence and clinical outcomes, especially bleeding events, were verified using local and Ministry of Health databases and through telephone follow-up. Patients were excluded from the study based on the following criteria:

- Presence of prosthetic heart valves or rheumatic mitral valve stenosis.
- Occurrence of bleeding events during follow-up or receipt of erythrocyte transfusions.
- Diagnosis of haematological or oncological diseases or severe hepatic dysfunction.
- Chronic kidney disease (CKD), defined as an estimated glomerular filtration rate (eGFR) of less than 50 mL/min/1.73 m^2^.
- Established chronic inflammatory conditions, malnutrition, or malabsorption syndromes.
- Use of vitamin K antagonists (VKAs) or combination therapy.
- Any modifications in medication regimen during the follow-up period.
- Mortality within the first year of follow-up.
- Incomplete data in the local database.

Patients with chronic inflammatory conditions and malabsorption syndromes were excluded because these conditions could independently affect haematological parameters, such as haemoglobin levels, regardless of DOAC therapy. Chronic inflammation can induce anaemia of chronic disease, and malabsorption syndromes may impair nutrient absorption critical for erythropoiesis. Excluding these patients ensured that changes in haematological parameters were attributable to DOAC therapy rather than confounding conditions.

Patients who passed away within the first year of follow-up were excluded to ensure that any potential haematological changes observed were associated with the effects of DOAC therapy in stable conditions rather than with acute clinical events or underlying severe conditions that may have led to mortality.

These patients were subsequently categorised into two groups based on the change in their haemoglobin (Hb) levels (<2 g/dL or ≥2 g/dL) after one year of treatment, compared to baseline values.

**Figure 1:**
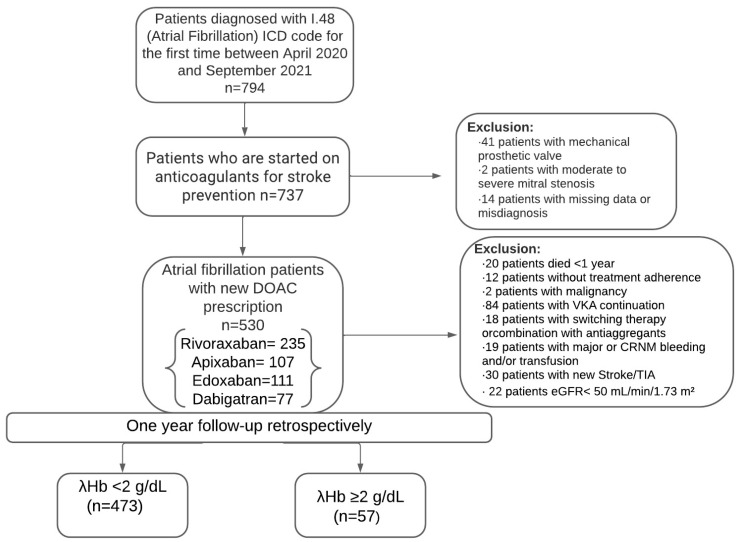
Flowchart of the study population.

The study flowchart is illustrated in Figure 1. The study protocol adhered to the ethical principles of the 1975 Declaration of Helsinki, and ethical approval was obtained from the local ethics committee.

### 2.1. Definitions

We employed precise definitions of various conditions to ensure uniformity and precision in the analysis. The definitions used are as follows:

Anaemia: According to the World Health Organization guidelines, anaemia is defined as an Hb concentration <12 g/dL for women and <13 g/dL for men [6].

Major Bleeding: Major bleeding is defined as any bleeding event that results in death or overt bleeding that leads to a decrease in Hb of at least 2 g/dL, requires the transfusion of two or more units of packed red blood cells, or occurs in a critical anatomical location such as the intracranial or retroperitoneal areas.

Clinically Relevant Nonmajor Bleeding (CRNMB): CRNMB events are identified as overt bleeding incidents that require medical intervention but do not meet the severity criteria of major bleeding.

Nuisance Bleeding: Nuisance bleeding was defined as minor bleeding episodes, including any bruising, haemorrhoidal bleeding, or other mild bleeding that did not require medical intervention. This category captures less severe bleeding events that may affect patient comfort and quality of life.

DOAC Reduced Dose: Administration of a DOAC at a dose below the standard or full therapeutic level based on pharmacological guidelines and clinical indications.

### 2.2. Statistical Analysis

Data were analysed on SPSS, the Statistical Package for Social Sciences (SPSS) for Windows (version 21.0; SPSS Inc., Chicago, IL, USA). Descriptive statistics were presented as median (Q1–Q3), frequency distributions, and percentages. Pearson’s chi-squared test and Fisher’s exact test were used to evaluate categorical variables. Since medians (IQR) were used for data presentation, it can be inferred that most variables did not follow a normal distribution. This was confirmed through normality tests (Kolmogorov–Smirnov test/Shapiro–Wilk test), with *p*-values indicating non-normal distribution for key variables. The *p*-values for these tests ranged from <0.05, supporting the choice of nonparametric statistical methods for analysis. The Mann–Whitney U test was used to evaluate the statistical significance between two independent groups for non-normally distributed variables. The Wilcoxon signed-rank test was used to compare the two dependent groups. In multivariate analysis, logistic regression analysis was conducted to examine independent predictors for a decrease in Hb (ΔHb ≥ 2 g/dL) using potential factors identified in previous analyses and multicollinearity diagnostics were performed. The Hosmer–Lemeshow test was used to assess the model fit. *p* values < 0.05 were regarded as statistically significant.

## 3. Results

Nineteen patients experienced major or CRNMB and/or required transfusions, and 39 patients had new stroke or TIA incidents during the follow-up period; these patients were excluded from the study.

The study included 530 patients with AF with a median age of 74 (interquartile range [IQR]: 67–80.25). The gender distribution was relatively balanced, enhancing the representativeness of the sample. Participants were categorised based on the specific DOAC regimen they received: rivaroxaban (*n* = 235), apixaban (*n* = 107), edoxaban (*n* = 111), and dabigatran (*n* = 77). No significant differences were noted in baseline demographic characteristics or comorbid conditions among these groups, which included metrics such as hypertension, diabetes mellitus, and prior myocardial infarction. However, only two parameters showed significant differences among the participant groups. A statistically significant age difference (*p* = 0.032) was observed between the rivaroxaban (73 (67–79)) and apixaban (76 (69–84)) groups.

Over the 12-month follow-up period, no significant difference was observed in the use of low-dose DOACs between the apixaban-edoxaban and rivaroxaban-dabigatran groups. The baseline characteristics, risk factors, and scoring for bleeding and ischaemic risk in the participants with AF are detailed in Table 1.

During the follow-up period, significant reductions were detected in Hb and Htc levels were observed in patients treated with rivaroxaban, edoxaban, and dabigatran. Specifically, patients on rivaroxaban experienced a median pretreatment Hb of 14.0 g/dL (IQR: 13.1–15.1), which decreased significantly to 13.5 g/dL (IQR: 12.6–14.7; *p* < 0.005). Edoxaban-treated patients exhibited a similar trend, with a reduction from 12.8 g/dL (IQR: 11.5–14.1) to 12.6 g/dL (IQR: 11.5–14.0; *p* = 0.026). In contrast, no significant changes in Hb or Htc levels were noted in the apixaban group, where median Hb levels remained stable (pretreatment: 13.2 g/dL [IQR: 12.0–14.5]; post-treatment: 13.3 g/dL [IQR: 11.8–14.5]; *p* = 0.817). A summary of these haematological changes is detailed in Table 2.

In analysing the characteristics of patients experiencing a clinically significant Hb decrease (≥2 g/dL) versus those who did not (<2 g/dL), we found no differences in terms of age, gender, or DOAC regimen. However, higher CHA2DS2-VASc (*p* = 0.019) and HAS-BLED (*p* = 0.013) scores were associated with the group experiencing a ≥2 g/dL decrease in Hb. Additionally, this group had a higher incidence of prior stroke or transient ischaemic attack (TIA), with 17.5% of patients (*n* = 10) affected compared to 8.8% (*n* = 42) in the group with a smaller Hb decrease (*p* = 0.038).

Baseline haematological parameters were also noteworthy. Patients with a significant Hb decrease had higher initial Hb values (14.6 g/dL [IQR: 13.1–15.7]) compared to those without a significant decrease (13.5 g/dL [IQR: 12.3–14.6]; *p* = 0.002). Similarly, initial Htc levels were higher in the ≥2 g/dL decrease group (42.9% [IQR: 39.2–45.7]) compared to the <2 g/dL group (40% [IQR: 36.5–43.2]; *p* = 0.001). These group characteristics and comparisons are shown in Table 3.

When evaluating parameters distinguishing Hb decrease, the baseline Hb value’s predictive power (C statistic: 0.63, 95% CI: 0.55–0.70) was higher than that of the CHA_2_DS_2_VASc score (C statistic: 0.59, 95% CI: 0.51–0.67) and HAS-BLED score (C statistic: 0.58, 95% CI: 0.50–0.66).

In the multivariate analysis conducted to evaluate parameters associated with Hb decrease (∆Hb ≥ 2 g/dL), the only statistically significant parameter was the pre-DOAC Hb value (OR: 1.424 [1.178–1.723], *p* < 0.005) (Table 4).

## 4. Discussion

Our study revealed that patients with AF undergoing DOAC therapy for stroke prevention experienced a decrease in Hb levels, even in the absence of clinically detectable events. This decrease in Hb levels was observed across the entire patient population, regardless of the treatment regimen and drug dosage, when confounding parameters were included in the analysis. We identified the baseline Hb value as a predictive parameter for a decrease in Hb (ΔHb ≥ 2 g/dL), with higher initial values correlating with more significant treatment-related reductions in Hb levels **(Central Illustration)**.

Numerous studies have focused on the impact of anaemia on cardiovascular outcomes. The potential adverse effects of anaemia have been investigated in various patient groups, including those with heart failure, coronary artery disease, and AF [7]. A meta-analysis by Kwok et al. revealed that anaemia in patients undergoing percutaneous coronary intervention was associated with an increased incidence of major adverse cardiovascular events (MACE), mortality, reinfarction, and bleeding [8]. The presence of anaemia in patients with heart failure not only worsens clinical outcomes but also diminishes their response to medical treatment [9,10]. In the context of AF, anaemia has been identified as an independent predictor of all-cause mortality and hospitalisation [11]. Krittayaphong et al. highlighted anaemia as an independent predictor of bleeding, heart failure, and all-cause mortality in 1562 patients with AF [12]. Kodani et al. also noted that an Hb level of <12 g/dL was an independent risk factor for all-cause mortality and composite events in Japanese patients with nonvalvular AF [7]. These findings underscore the serious clinical challenges associated with anaemia. Although literature predominantly presents data on conditions involving bleeding, information on stable patients is scarce. Our study, which followed a patient population without serious events, detected a >2 g/dL decrease in Hb in a subset of patients, despite the average pre-DOAC and post-DOAC Hb levels remaining within normal limits (13.6 g/dL to 13.3 g/dL). However, our study uniquely highlights the occurrence of a significant decrease in Hb in a stable patient population without serious events, emphasising the need for careful management of such reductions to mitigate their negative impact on cardiovascular outcomes.

The differential impact of DOACs on haemoglobin levels observed in our study may align with findings from recent real-world data and clinical trials. The Italian Registry (IRIS), the largest dataset evaluating catheter ablation with rivaroxaban in Italy, concluded that rivaroxaban demonstrated safety and efficacy in clinical practice [13]. Similarly, the ELIMINATE-AF trial established that uninterrupted edoxaban therapy is a viable alternative to vitamin K antagonists in patients undergoing atrial fibrillation ablation, supporting its safety profile [14]. These findings reinforce the idea that while rivaroxaban and edoxaban have proven efficacy, specific haematological changes might still occur under certain clinical circumstances. Our results underscore the importance of careful monitoring in these patient cohorts to manage potential subclinical decreases in haemoglobin levels effectively.

Our data shows that this phenomenon occurs independently of the type and dosage of DOACs in patients with ΔHb ≥ 2 g/dL. Although there was a consistent trend between stroke and bleeding risk scores and a decrease in Hb level, this did not reach statistical significance, possibly because the study population’s average ischaemic and bleeding risk scores were not excessively high (CHA_2_DS_2_VASc scale 4(3–5), HAS-BLED index 2 (2–3)).

Interestingly, our findings uniquely identified pre-DOAC Hb value as the sole predictor of a ΔHb ≥ 2 g/dL, with higher baseline Hb levels associated with greater decreases. This ‘paradoxical’ observation has been reported previously. Jaffery et al. found that chronic kidney disease (eGFR < 60 mL/min) and obesity (BMI > 30) were predictors of a subclinical >1 g/dL decrease in Hb levels following percutaneous coronary and peripheral procedures [15]. Brener et al. identified that many patients who developed events in the context of the acute coronary syndrome had baseline Hb values above the anaemia threshold (>14 g/dL), with the majority of patients in the nonanaemia group experiencing a >4 g/dL decrease in Hb levels without significant bleeding [16]. A similar ‘paradoxical’ finding was reported by Sairaku et al., who observed a >2 g/dL decrease in Hb in patients with nonanaemic AF following ablation procedures. This was explained by the potential of pre-existing anaemia to cause a hypercoagulable state and/or operators being more cautious in avoiding blood loss in patients with pre-existing anaemia [17].

The paradoxical observation that patients with higher baseline haemoglobin (Hb) levels experienced a greater decrease prompted further investigation. Although similar phenomena have been reported in the literature, the underlying mechanisms remain elusive. One potential reason could be the relatively stable condition of these patients, leading to less frequent clinical follow-ups and the potential for delayed recognition of subclinical issues. Additionally, patients and/or clinicians may have overlooked nuisances and minor bleeding that did not warrant immediate attention.

Another possible explanation is that patients with higher baseline Hb may undergo greater haemodilution or experience a more pronounced haematological adaptation when starting anticoagulant therapy. These patients might also have a larger margin for Hb reduction before reaching clinical thresholds, increasing their risk of unnoticed subclinical bleeding. Furthermore, factors such as fluid retention or predisposing conditions could contribute to this trend.

The finding that baseline Hb is the sole predictor of significant decreases highlights the complexity of managing patients on DOAC therapy and reinforces the importance of individualised patient monitoring. Careful, proactive follow-up is particularly crucial for patients with high baseline Hb to detect and manage potential subclinical haemoglobin decreases before they impact patient outcomes.

The identification of baseline Hb as a predictor of significant decreases has important clinical implications. Clinicians managing patients on DOAC therapy should be particularly vigilant when treating those with higher baseline Hb levels. These patients may require closer monitoring for potential subclinical Hb declines, even if they initially present as clinically stable. This finding suggests that routine haematological evaluations should be considered more frequently for this subset of patients to preemptively detect any significant changes and mitigate adverse outcomes.

Practical strategies could include scheduling periodic follow-up blood tests at shorter intervals and advising patients on recognising and reporting subtle symptoms indicative of subclinical bleeding. Tailored patient education and monitoring protocols could help in managing individuals at higher risk and ensuring that reductions in Hb do not progress to clinical anaemia or other complications. 

### Limitations

This study has several limitations that should be acknowledged. The retrospective design inherently limited the ability to control for all potential confounding factors. Despite meticulous efforts to account for known variables influencing Hb levels, undiagnosed secondary conditions, such as malignancies, remain a potential limitation. Additionally, the study was conducted at a single centre, which may restrict the generalisability of the findings to broader populations. The relatively small sample size, although sufficient for detecting significant changes, may affect the robustness of subgroup analyses. The follow-up period was confined to twelve months, limiting the study’s ability to capture long-term clinical scenarios that might contribute to observed decreases in Hb levels. Furthermore, it is important to note that a percentage analysis of Hb changes was not feasible with the current dataset, potentially influencing the interpretation of the greater absolute decreases seen in individuals with higher baseline Hb levels. This type of analysis was also not reported in the studies cited in our discussion. Recognising these limitations is crucial for interpreting our findings and guiding future multicentre studies with larger sample sizes, extended follow-up periods, and more comprehensive data analyses.

## 5. Conclusions

This study identified baseline haemoglobin (Hb) levels as a significant predictive factor for a decrease in Hb ≥ 2 g/dL among patients receiving DOAC therapy, with higher initial Hb levels being associated with more substantial reductions. This pattern was observed consistently across various DOAC types and dosages. These findings underscore the importance of individualised patient monitoring, particularly for those presenting with higher baseline Hb, to detect subclinical changes and preempt potential complications. Implementing tailored surveillance and management strategies based on these insights may enhance patient safety and optimise clinical outcomes.

## Figures and Tables

**Table 1:** Baseline demographic, clinical, and laboratory evaluations.

	Overall (*n* = 530)	Rivaroxaban (*n* = 235)	Apixaban (*n* = 107)	Edoxaban (*n* = 111)	Dabigatran (*n* = 77)	*p*
Age, years	74 (67–80)	73 (67–79)	76 (38–100)	73 (65–80)	74 (65.5–80.5)	**0.032**
Gender, *n* (%)						
Male, *n* (%)	240 (%45.3)	99 (%42.1)	52 (%48.6)	48 (%43.2)	41 (%53.2)	0.311
Female, *n* (%)	290 (%54.7)	136 (%57.9)	55 (%51.4)	63 (%56.8)	36 (%46.8)	
CHA_2_DS_2_-VASc	3.43 ± 1.36	3 (2–4)	3 (3–4)	3 (2–4)	4 (3–5)	0.419
HAS-BLED	1.88 ± 0.86	2 (1–2)	2 (1–2)	2 (1–3)	2 (1–2)	0.388
DM, *n* (%)	100 (%18.9)	36 (%15.3)	15 (%14)	30 (%27)	19 (%24.7)	0.18
HT, *n* (%)	439 (%82.8)	184 (%78.3)	89 (%83.2)	103 (%92.8)	63 (%81.8)	0.11
Prior MI, *n* (%)	90 (%17)	34 (%14.5)	21 (%19.6)	23 (%20.7)	12 (%15.6)	0.425
Previous Stroke/TIA, *n* (%)	52 (%9.8)	18 (%7.7)	12 (%11.2)	13 (%11.7)	9 (%11.7)	0.527
PAD, *n* (%)	20 (%3.8)	10 (%4.3)	1 (%0.9)	5 (%6.5)	4 (%3.6)	0.251
Congestive HF, *n* (%)	143 (%27)	54 (%23)	29 (%27.1)	27 (%35.1)	33 (%29.7)	0.18
eGFR, mL/min/1.73 m^2^	76 (59.3–88.3)	78.5 (63.8–89)	67 (50–83.5)	75 (61.1–90.5)	80 (60–89)	0.267
Abnormal Liver Functions, *n* (%)	2 (%0.4)	0	1 (%0.9)	0	1 (%1.3)	0.269
History of Bleeding, *n* (%)	42 (%7.9)	13 (%5.5)	10 (%9.3)	11 (%9.9)	8 (%10.4)	0.337
NSAID Use, *n* (%)	80 (%15.1)	30 (%12.8)	18 (%16.8)	17 (%15.3)	15 (%19.5)	0.493
DOAC Dose (Reduced)	143 (%27)	55 (%23.4)	43 (%40.2)	22 (%19.8)	45 (%58.4)	**<0.005**

Quantitative variables are specified as medians (Q1–Q3). Categorical variables are presented as numbers and percentages. DM: diabetes mellitus; HT: hypertension; MI: myocardial infarction; TIA: transient ischemic attack; PAD: peripheral artery disease; NSAID: Nonsteroidal anti-inflammatory drug; DOAC, direct oral anticoagulant.

**Table 2:** Laboratory Parameters of Patients Pre- and Post-DOAC.

	Rivoraxaban	*p*	Apixaban	*p*	Edoxaban	*p*	Dabigatran	*p*
Hb, g/dL								
Pre-Doac	14 (13.1–15.1)	**<0.005**	13.2 (12–14.5)	0.817	12.8 (11.5–14.1)	**0.026**	13.7 (12.5–15.2)	**<0.005**
Post-Doac	13.5 (12.6–14.7)		13.3 (11.8–14.5)		12.6 (11.5–14)		12.9 (11.9–14.6)	
Htc, %								
Pre-Doac	41.2 (38.6–43.8)	**<0.005**	39.2 (35.7–42.3)	0.663	39.4 (35–43.1)	**0.031**	40.3 (37.5–44.5)	**<0.005**
Post-Doac	39.5 (37.1–42.9)		39 (35.1–42.3)		38.7 (35.2–41.7)		38.3 (35.2–42.3)	
Plt, 10^3^/uL								
Pre-Doac	237 (196–280)	0.882	216 (187–271)	0.598	227 (180–279)	0.269	226 (181–281)	0.28
Post-Doac	242 (191–284)		221 (175–273)		215 (178–274)		238 (201.5–277)	

Quantitative variables were specified as medians (Q1–Q3). Categorical variables were shown as numbers and percentage values. DOAC: direct oral anticoagulants; Hb: hemoglobin; Htc: haematocrit; Plt: platelets.

**Table 3:** Baseline parameters according to ∆Hb.

	∆Hb < 2 (*n* = 473)	∆Hb ≥ 2 (*n* = 57)	*p*
Age, years	74 (67–81)	75 (66–80)	0.696
Gender, *n* (%)			
Male, *n* (%)	214 (%40.3)	26 (%4.9)	0.958
Female, *n* (%)	259 (%48.8)	31 (%5.8)	
CHA2DS2-VASc scale	3 (2–4)	4 (3–5)	**0.019**
HAS-BLED index	2 (1–2)	2 (2–3)	**0.013**
DM, *n* (%)	2 (1–3)	2 (1–3)	0.655
HT, *n* (%)	390	49	0.506
Prior MI, *n* (%)	79 (%14.9)	11 (%2)	0.622
Previous Stroke/TIA, *n* (%)	42	10	**0.038**
PAD, *n* (%)	16	21	0.158
Congestive HF, *n* (%)	122	21	0.076
Abnormal Liver Functions, *n* (%)	2	0	0.623
History of Bleeding, *n* (%)	36	6	0.29
NSAID Use, *n* (%)	72	8	0.813
DOAC Type, *n* (%)			
Rivaroxaban	211	24	0.232
Apixaban	99	8	
Edoxaban	99	12	
Dabigatran	64	13	
DOAC Dose (Reduced), *n* (%)	126	17	0.609
Hb, g/dL			
Pre-doac	13.5 (12.3–14.6)	14.6 (13.1–15.7)	**0.002**
Post-doac	13.4 (12.3–14.6)	11.4 (10.2–12.8)	**<0.005**
	*p* = 0.167	***p* < 0.005**	
Htc, %			
Pre-doac	40 (36.5–43.2)	42.9 (39.2–45.7)	**0.001**
Post-doac	39.7 (36.7–42.9)	34.8 (30.7–37.9)	**<0.005**
	*p* = 0.245	***p* < 0.005**	
Plt, 10^3^/uL			
Pre-doac	227 (187.5–281)	232 (194.5–260.5)	0.726
Post-doac	233 (185–277)	237 (201–284)	0.386
	*p* = 0.467	*p* = 0.831	
eGFR, mL/min/1.73 m^2^			
Pre-doac	76 (59.3–88.3)	76 (62.5–89)	0.496
Post-doac	71.7 (54–85)	69 (54.1–85.2)	0.802
	*p* = 0.654	*p* = 0.345	

Quantitative variables were specified as medians (Q1–Q3). Categorical variables were shown as numbers and percentage values. CHA2DS2-VASc: congestive heart failure, hypertension, age ≥ 75 years (doubled), diabetes mellitus, prior stroke, transient ischemic attack, or thromboembolism [doubled], vascular disease, age 65–74 Years, sex category; HAS-BLED: hypertension, abnormal renal/liver function, stroke, bleeding history or predisposition, labile international normalized ratio, elderly, drugs/alcohol concomitantly. DM: diabetes mellitus; HT: hypertension; MI: myocardial infarction; TIA: transient ischemic attack; PAD: peripheral artery disease; HF: heart failure; NSAID: nonsteroidal anti-inflammatory drugs; DOAC: direct oral anticoagulants; Hb: haemoglobin; Htc: haematocrit; Plt: platelets; eGFR: estimated glomerular filtration rate.

**Table 4:** Associations between key patient characteristics and decrease in haemoglobin (∆Hb) during follow-up.

	OR (%95 CI)	*p*
Age	0.998 (0.961–1.036)	0.900
Gender	1.434 (0.743–2.767)	0.283
CHA_2_DS_2_-VASc scale	1.204 (0.882–1.645)	0.243
HAS-BLED index	1.418 (0.895–2.248)	0.137
Pre-DOAC Hb	1.424 (1.178–1.723)	**<0.005**
Pre-DOAC eGFR	1.009 (0.994–1.024)	0.260
DOAC Dose (Reduced)	0.985 (0.453–2.138)	0.969

CI, confidence interval; OR, odds ratio; DOAC, direct oral anticoagulant; Hb, haemoglobin; eGFR, estimated glomerular filtration rate. Variance inflation factors (VIFs) were calculated for key variables, including eGFR and DOAC dose, and all VIFs were found to be below the commonly accepted threshold of 5.

## Data Availability

The datasets generated and analyzed during the current study are available from the corresponding author upon reasonable request. Access to the data is granted to researchers who are interested in replicating or further investigating the findings of this study, in accordance with applicable ethical guidelines and privacy regulations.
